# Temporomandibular Joint Ankylosis in a Three-Year-Old Female: Anaesthesia, Airway Management, and Mandibular Distraction Osteogenesis

**DOI:** 10.7759/cureus.60828

**Published:** 2024-05-22

**Authors:** Amreesh Paul, Anjali Borkar, Dnyanshree Wanjari

**Affiliations:** 1 Anaesthesiology, Jawaharlal Nehru Medical College, Datta Meghe Institute of Higher Education and Research, Wardha, IND

**Keywords:** mandibular distraction osteogenesis, difficult airway management, pediatric difficult airway, pediatric anesthesia, temporomandibular joint ankylosis

## Abstract

Temporomandibular joint (TMJ) ankylosis is generally characterised by a complex aetiology, with several contributing causes, including infections, autoimmune diseases, trauma, and congenital anomalies. This case report describes a three-year-old female suffering from traumatic temporomandibular ankylosis with retrognathia, severe mouth-opening restriction, and obstructive sleep apnea (OSA). The present case highlights the difficulties with TMJ ankylosis, especially when access to healthcare is sought out late and delayed diagnosis is prevalent. Mandibular distraction osteogenesis and awake fiberoptic intubation were used in the surgical and anaesthetic management of this case, with the otorhinolaryngology team on standby to perform a tracheostomy if required, highlighting the necessity of a multidisciplinary approach in such cases. Patients with TMJ ankylosis have significant life-altering changes, including psychological stress, chewing difficulty, speech difficulties, facial distortion, and speech impediment. When OSA progresses, it also presents more health risks. For the purpose of treating TMJ ankylosis, avoiding serious problems, and enhancing patient well-being, prompt diagnosis and therapy are crucial. In order to optimise patient results, this case study highlights the need for knowledge and research in the treatment of TMJ ankylosis as well as the requirement of medical professionals working together in a synergistic way.

## Introduction

The temporomandibular joint (TMJ) is a synovial joint between the mandibular condyle and the articular fossa of the temporal bone, involved in speech and mastication. It comprises several structures, including the glenoid fossa of the temporal bone, mandibular condylar head, articular disc, muscles, and ligaments [1]. The calcification of ligaments or fusion of bones around this joint causes restricted joint movement, known as ankylosis [2]. Trauma is the leading cause of TMJ Ankylosis, while other causes include congenital malformations, ear infections, local/systemic infections, auto-immune disorders, forceps delivery, and unknown aetiology [3]. TMJ ankylosis is commonly seen in patients with congenital anomalies like Treacher-Collins syndrome, Pierre-Robin syndrome, and Apert syndrome. Traumatic TMJ ankylosis is an increasing trend in developing countries, as mandibular condyle fractures are often overlooked and are diagnosed only when the patient comes in with ankylosis [4]. Ankylosis of the TMJ leads to difficulty in chewing, decreased oral intake, reduced mouth opening, impairment of speech, distortion of facial features, retrognathia, micrognathia, and stress. This results in obstructive sleep apnea (OSA) in longstanding cases as the oral and pharyngeal areas are decreased significantly. These factors usually make surgical intervention mandatory [5-7]. In developing countries, people from remote areas find it difficult to access healthcare and generally do not seek medical care until the problem hampers one’s lifestyle. Hence, most patients are encountered in the hospital at advanced stages where mouth opening is almost nil [8]. Surgical methods to increase mouth opening are done to treat TMJ Ankylosis. These include TMJ arthroplasty, autogenous costochondral rib graft, distraction osteogenesis followed by extensive mouth-opening exercise, corrective orthognathic surgery, total joint reconstruction, or prosthesis usage [9]. Airway management and ventilation become significantly difficult due to a combination of muscle weakness, obstruction of the pharyngeal and hypopharyngeal lumens, and sub-atmospheric intra-pharyngeal pressure [10]. Airway management in these cases includes nasal intubation (by blind technique or using a fiberoptic bronchoscope), retrograde intubation, and tracheostomy [4]. This is a report of a case of a three-year-old female with traumatic temporomandibular ankylosis, associated retrognathia, nil mouth opening, obstructive sleep apnea, and subsequent anaesthetic management for mandibular distraction osteogenesis.

## Case presentation

**Figure 1:**
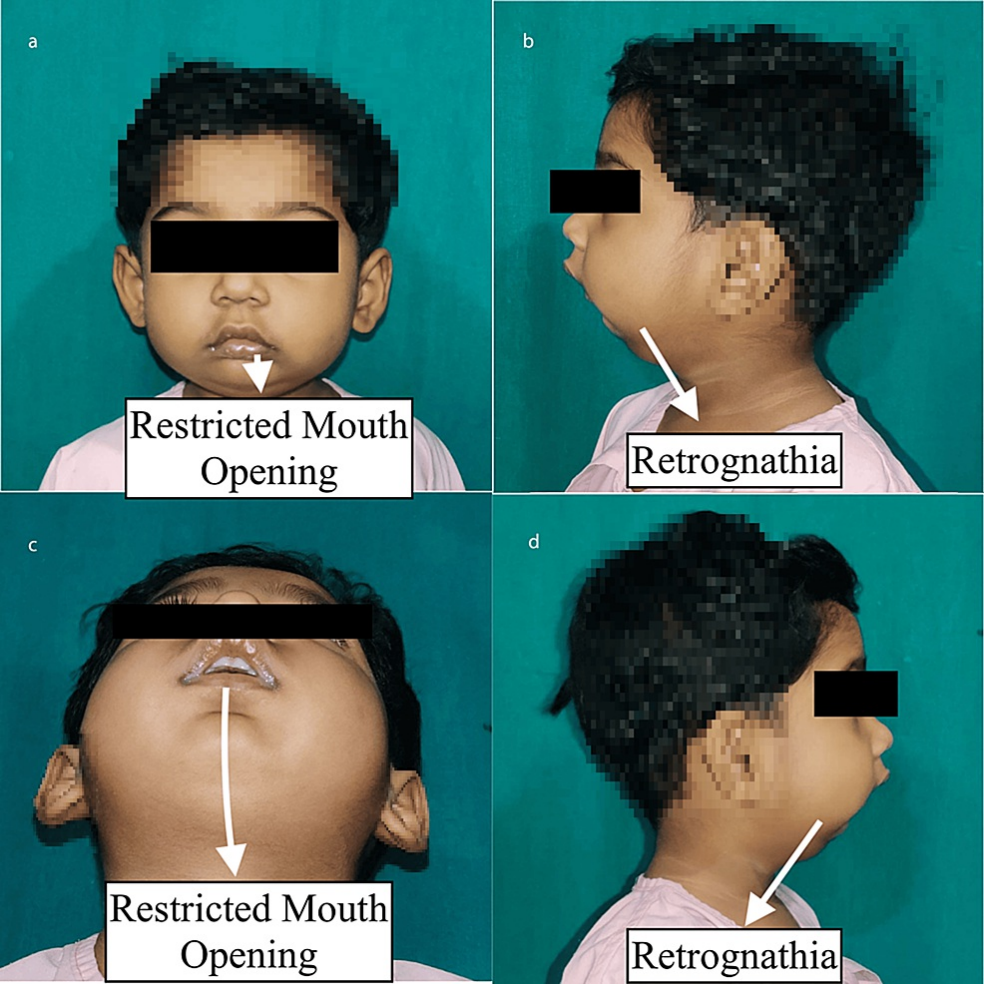
Pre-operative image showing restricted mouth opening and retrognathia Figures a, c - Showing restricted mouth opening; Figures b, d - Showing retrognathia.

A three-year-old female was brought to our hospital by her parents with complaints of decreased oral intake due to absent mouth opening, as described by the mother. The patient was also malnourished as a result of this. The patient was normal till a year back when she had pus discharge and associated swelling over the right pre-auricular region suggestive of the pre-auricular abscess. The abscess was drained, and the patient started developing these symptoms. The patient also had a small and indrawn lower jaw, as seen in Figure 1. The mother also informed that the patient had episodes of sudden arousal from sleep during the night. Given these symptoms, the patient was planned to undergo distraction osteogenesis of the bilateral temporomandibular joint.

A detailed pre-anaesthetic evaluation was done. The patient weighed 10 kg, with a height of 84 cm and a Z score of -1.1. She was a full-term normal delivery. All developmental milestones have been achieved to date. She has received all her immunisations to date. The mother gave a history of difficulty in mastication since the incision and drainage. The patient had undergone the incision and drainage under general anaesthesia with no reported complications. The child had a history of multiple episodes of waking abruptly from sleep daily. Pulse rate was 124/min; blood pressure was 100/60mmHg; the respiratory rate was 24/min; and room air saturation was 97%. Airway assessment revealed nil mouth opening. Systemic examination had no significant findings. Blood investigations, chest X-rays, electrocardiogram, and echocardiogram were all within normal limits. The oxygen saturation of the patient during sleep constantly fell to about 80% due to OSA. The child’s parents were explained about general anaesthesia, its benefits, risks, and complications. Informed and written consent for the same was obtained.

**Figure 2:**
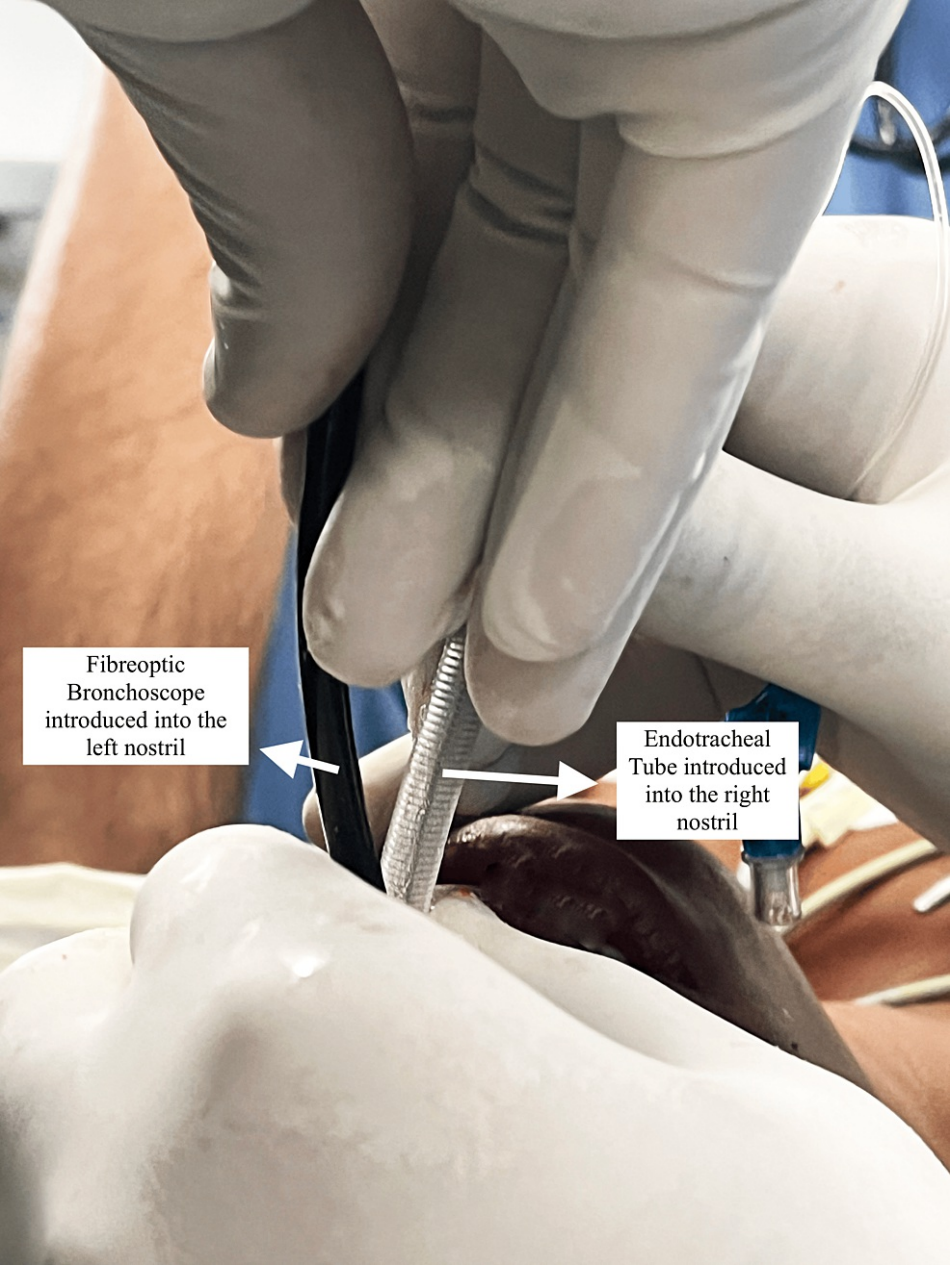
Intra-operative image A fiberoptic bronchoscope was introduced into the left nostril, and a flexometallic endotracheal tube was introduced into the right nostril.

**Figure 3:**
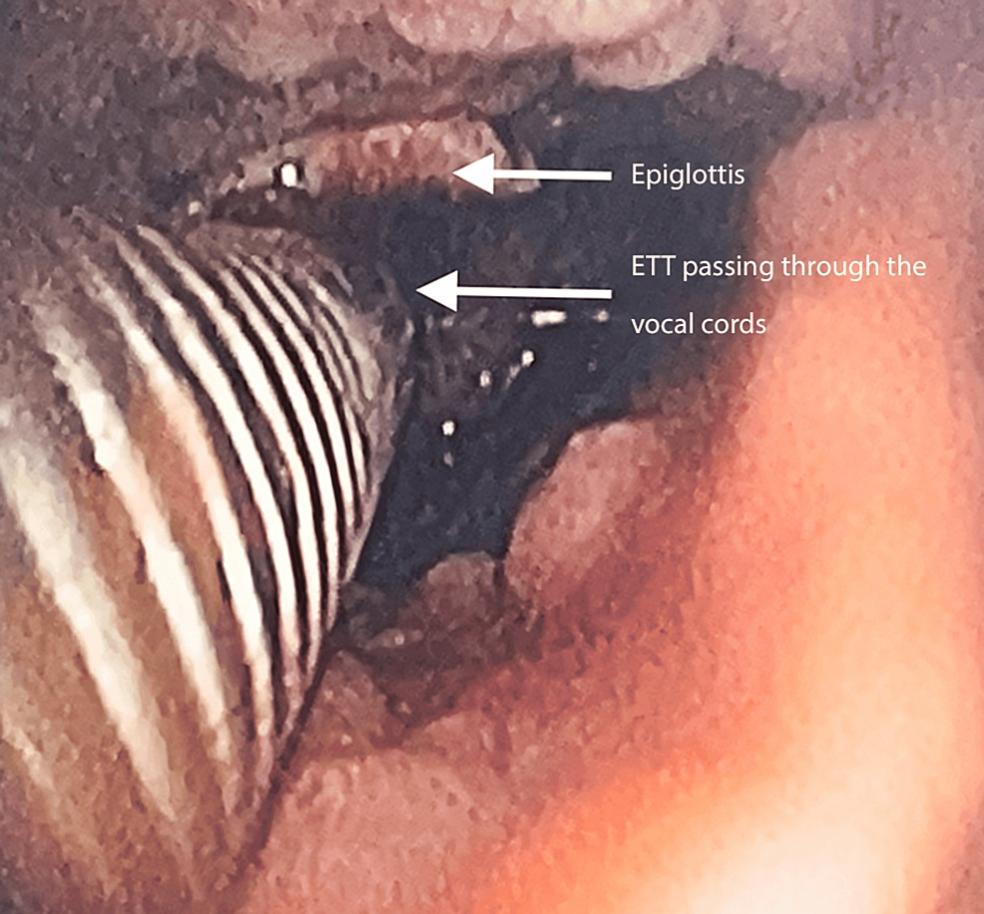
Fiberoptic view of the glottis ETT: Endotracheal tube The image shows the reinforced flexometallic endotracheal tube in situ.

On the day of the surgery, the patient was kept six hours nil per os for solids and two hours nil per os for liquids. She was shifted to the pre-operative room and was nebulised with 5 ml of lignocaine 4%. The patient was then shifted to the operation theatre, non-invasive multipara monitors as per American Society of Anesthesiology (ASA) standards were attached, and baseline vital parameters were noted. The patient was given oxygen, nitrous oxide, and sevoflurane 2% through bag-mask ventilation. A 24-gauge intravenous cannula was inserted in the left upper limb. She was pre-medicated with intravenous glycopyrrolate 0.04 mg, midazolam 0.5 mg, and fentanyl 20 mcg. The otolaryngologist team was kept on standby, in case emergency tracheostomy was required to secure the airway or in case of accidental extubation. Superior laryngeal nerve and transtracheal blocks were given with lignocaine 4% using the landmark technique. The smallest fiberoptic bronchoscope available at our Institute had an internal diameter of 4 mm. Hence, railroading it with a flexometallic endotracheal tube with an internal diameter of 4.5 mm was impossible. With the child breathing spontaneously, the fiberoptic bronchoscope was introduced into the left nostril and advanced until the vocal cords were in sight. Following this, a cuffed reinforced flexometallic endotracheal tube of internal diameter 4.5 mm was introduced into the right nostril and was advanced to the vocal cords, as seen in Figure 2. Under the vision of the fiberoptic bronchoscope, the endotracheal tube was manipulated into the trachea by giving neck flexion. The tube position was confirmed by fiberoptic visualisation, five-point auscultation, and capnography, as seen in Figure 3.

Following this, the surgery was commenced. The patient was maintained on sevoflurane, a mixture of oxygen and nitrous oxide (FiO2-50%) and atracurium. The patient was prepared and draped according to standard surgical protocols. Distractor placement was done over the left and right sides, and closure was done in layers. Pressure dressing was given over the bilateral pre-auricular region.

**Figure 4:**
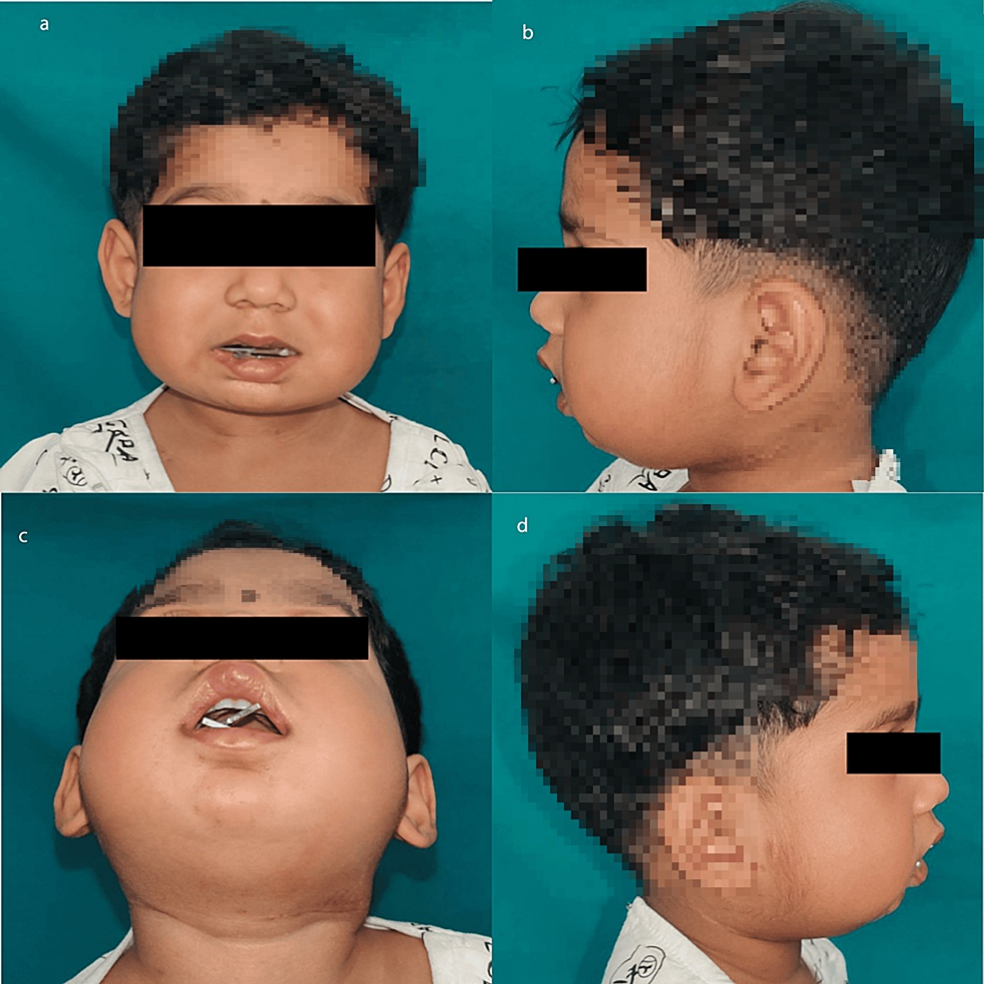
Post-operative images after distraction Images a, b, c, and d show improved mouth opening.

The patient was reversed, extubated, and shifted to the surgical intensive care unit for further monitoring. In the postoperative period, a total distraction of 11mm was done over a week, as seen in Figure 4. The patient was discharged on postoperative day 10. The patient was followed up on an outpatient basis. The patient’s mother revealed that the patient had decreased episodes of breathlessness and arousal from sleep at night. Saturation monitoring revealed no drop less than 93%.

## Discussion

TMJ ankylosis poses a wide variety of anaesthetic and surgical challenges, and the complexity is due to a variety of factors, especially in pediatric cases [3]. The presence of bilateral TMJ ankylosis can make the airway difficult due to the inability to visualize the vocal cords due to restricted mouth opening. Issues such as obstructive sleep apnea and retrognathia tend to complicate these issues [5]. A great difference is seen in anatomy and physiology between children and adults, and these tend to diminish by 10 to 12 years of age. These disparities have very important consequences while administering anaesthesia, and hence children of young age require precise planning for anaesthesia, including drug dosing and techniques used for airway maintenance [11].

Pediatric difficult airway has been a significant cause of increased morbidity and mortality in children undergoing surgical procedures under anaesthesia. Improper planning of difficult airway management may lead to various complications, such as cardiac arrest, hypoxic brain injury, and even death [12].

If the planned airway management fails, the anaesthesiologists must have a backup plan, such as an emergency tracheostomy, emergency cricothyroidotomy, or vascular access for extracorporeal membrane oxygenation [13]. Supraglottic airways are generally used as a rescue device, but in our case, they could not be used as the mouth opening was restricted [14]. The utilization of fiberoptic bronchoscopy is the gold standard for establishing the airway in difficult pediatric airway management [15].

Generally, fiberoptic nasal intubation is achieved by railroading the endotracheal tube over the bronchoscope. Owing to the unavailability of a pediatric fibreoptic bronchoscope, we chose to use an adult fibreoptic bronchoscope inserted through one nostril to visualise the vocal cords and guide the endotracheal tube in through the other nostril while the child was awake as done by Sharma et al. [5]. The otolaryngorhinology team was on standby in case of failure to intubate and accidental extubation. Managing the airway in a pediatric case is always difficult, and it is important to provide adequate sedation without compromising ventilation. Postoperative stridor and respiratory depression were anticipated, and the child was monitored meticulously in the postoperative period. The successful management of this case through mandibular distraction osteogenesis exemplifies the importance of a multidisciplinary approach involving oral and maxillofacial surgeons, anaesthesiologists, and other healthcare professionals.

## Conclusions

In conclusion, the case of this three-year-old child with traumatic TMJ ankylosis, retrognathia, OSA, and severe mouth-opening restriction emphasises the intricate nature of TMJ ankylosis and its wide-ranging effects. This condition presents major challenges because of its vast range of etiological causes. Anesthesiologists, surgeons, and other medical professionals must work together to achieve good outcomes for the patient, as demonstrated by the effective management of this case. TMJ ankylosis has serious consequences, including OSA, facial deformities, nutritional problems, speech difficulties, and psychological instability. To prevent serious consequences and enhance patients' overall health and functioning, prompt diagnosis and management are crucial. This case report highlights the anaesthetic technique used, the need for proper planning and improvisations, the importance of interdisciplinary teamwork as well as the urgent need for research and educational initiatives on the subject of treating TMJ ankylosis. Through a holistic approach to managing the airway in such cases, we can enhance the quality of life for individuals impacted by this complex condition and strive towards improved results.
